# ARS-GS: Anisotropic Reflective Spherical 3D Gaussian Splatting

**DOI:** 10.3390/jimaging12040170

**Published:** 2026-04-15

**Authors:** Chenrui Wu, Xinyu Shi, Zhenzhong Chu, Yao Huang

**Affiliations:** Department of Mechanical Engineering, University of Shanghai for Science and Technology, Shanghai 200093, China; shixinyu@usst.edu.cn (X.S.); chuzhenzhong@usst.edu.cn (Z.C.); huangyao@usst.edu.cn (Y.H.)

**Keywords:** novel view synthesis, radiance fields, 3D Gaussians, reflective surfaces

## Abstract

3D scene reconstruction serves as a fundamental technology with widespread applications in virtual reality, structural inspection, and robotic systems. While recent advances in 3D Gaussian Splatting have significantly enhanced scene reconstruction capabilities, the performance of such methods remains suboptimal when applied to highly reflective environments. To overcome this limitation, we introduce ARS-GS, a novel framework that integrates Anisotropic Spherical Gaussian reflection modeling and spherical harmonics diffuse approximation into a physically based rendering pipeline. This architecture incorporates a skip connection between the Anisotropic Spherical Gaussian module and the Gaussian primitives, effectively preserving surface details while maintaining computational efficiency. Comprehensive experimental evaluations validate the efficacy of ARS-GS across multiple datasets. Specifically, our method establishes new state-of-the-art quantitative benchmarks, achieving a peak signal-to-noise ratio of 38.30 and a structural similarity index measure of 0.997 on the neural radiance fields synthetic dataset, alongside a peak signal-to-noise ratio of 46.31 on the Gloss Blender dataset. Furthermore, on the challenging reflective neural radiance fields real-world dataset, our approach secures the highest peak signal-to-noise ratio scores, highlighted by a metric of 26.26 on the Sedan scene. The proposed method also substantially reduces perceptual errors, yielding a learned perceptual image patch similarity as low as 0.204, thereby consistently outperforming existing techniques in the reconstruction of highly specular surfaces with superior geometric fidelity.

## 1. Introduction

Geometric multiview reconstruction, a fundamental paradigm in computer vision and computer graphics, focuses on extracting three-dimensional structures from sequential image data. This cornerstone technology serves as the driving force behind advancements in Simultaneous Localization and Mapping (SLAM) systems, autonomous navigation platforms, and digital content creation pipelines, among other applications. In recent years, neural radiance fields (NeRF) [1] have achieved impressive results in 3D reconstruction tasks using their neural network-based volumetric representation approach. Recent research has improved geometric reconstruction through signed distance field (SDF) integration [2,3,4], while advanced illumination models [5,6] address the rendering of complex specular reflections. However, NeRF remains computationally intensive, which significantly limits its practical deployment in interactive real-time applications.

3D Gaussian Splatting (3DGS) [7] marks a significant advance by proposing a fundamentally different rendering approach through learnable 3D Gaussian parameters, enabling efficient generation of high-resolution scenes. To improve geometric accuracy, several researchers [8,9,10] have proposed different types of geometric regularization. However, these methods face significant challenges in complex scenarios, particularly in accurately representing surfaces with high reflectance properties. The primary limitation stems from the absence of physically based lighting models in the current framework, leading to unreliable surface reconstruction in scenes with complex illumination. Although recent work by [11] has made progress in introducing an improved color representation paradigm to model high-frequency radiance variations in specular scenes, the lack of physical constraints in the rendering process still results in geometrically implausible artifacts. Therefore, developing reconstruction techniques that can effectively balance representational power and computational efficiency while maintaining physical accuracy in complex lighting scenarios remains a crucial challenge.

In this study, we present a comprehensive theoretical analysis of Anisotropic Spherical Gaussian (ASG) [12], establishing its mathematical correspondence with the specular component of the Bidirectional Reflectance Distribution Function (BRDF) model. Building upon this foundation, we introduce ARS-GS, a novel framework that seamlessly integrates ASG and spherical harmonics (SH) through physically based rendering (PBR) equations. ASG is used to model specular reflectance, while SH is used to represent diffuse components. For each Gaussian primitive, we incorporate sophisticated material properties that govern the interaction between ASG and SH components, enabling a comprehensive representation of view-dependent color characteristics.

Moreover, we enhance the performance of the framework by implementing a skip connection-like structure that enables direct gradient propagation from ASG module to GS positions, thereby accelerating coordinate convergence and improving reconstruction accuracy in highly reflective scenes.

In summary, our main contributions are as follows. In light of these challenges, this study aims to address the following research questions (RQs):

- RQ1: Can Anisotropic Spherical Gaussian (ASG) be effectively integrated with spherical harmonics (SH) within a physically based rendering (PBR) framework to accurately model complex specular and diffuse reflections in 3D Gaussian Splatting?
- RQ2: Does the introduction of a skip connection-inspired architecture between the ASG network and Gaussian primitives improve the optimization stability and geometric reconstruction fidelity of highly reflective surfaces?
- RQ3: How does the proposed ARS-GS framework perform compared to state-of-the-art novel view synthesis methods in terms of rendering quality and geometric accuracy across diverse and challenging datasets?

## 2. Related Work

### 2.1. Neural Radiance Fields (NeRF)

NeRF [1], as a groundbreaking deep learning-based 3D reconstruction method, has achieved significant progress in novel view synthesis (NVS) through implicit representation and volumetric rendering techniques. By leveraging Multi-Layer Perceptrons (MLPs) to model scene geometry and radiance fields, NeRF utilizes the volumetric rendering equation and MLPs’ inherent continuity to generate high-precision scene representations from sparse multiview images, establishing itself as the state-of-the-art method for photorealistic view synthesis. Building upon this foundation, researchers have developed numerous extensions to enhance NeRF’s capabilities across various aspects. For view synthesis optimization, Mip-NeRF [13,14] addressed the challenge of multiscale rendering by introducing techniques based on conical frustums, significantly improving rendering quality across various scene scales, particularly in distant viewpoints and wide-angle scenes. To further improve geometric accuracy, several methods have focused on combining surface reconstruction with radiance field modeling. Notable works [2,4,15,16,17,18] achieve more precise reconstruction by integrating implicit surface representations with NeRF, enabling joint optimization of geometry and appearance. The flexibility of NeRF has enabled significant advances in scene understanding and optimization. Recent developments have focused particularly on the optimization of camera parameters, with methods such as [19,20,21,22], demonstrating substantial improvements in the estimation and refinement of the camera pose. Furthermore, to address practical limitations in data acquisition, several efficient learning extensions of NeRF [23,24,25] have successfully demonstrated the ability to maintain high rendering quality while significantly reducing the required number of training images. However, despite these advances, NeRF-based methods face fundamental performance limitations due to their reliance on MLP queries along viewing rays. The need for hundreds of network evaluations per ray results in slow rendering and high memory consumption, further limiting their use in real-time scenarios.

### 2.2. 3D Gaussian Splatting (3DGS)

Recent advances in neural rendering have introduced novel scene representations beyond traditional NeRF. Ref. [7] presented a significant breakthrough by introducing 3D Gaussians as a scene representation, employing a tile-based fast differentiable rasterizer for image rendering. By representing each scene point as a 3D Gaussian distribution with directional expansion, this method improved rendering quality and computational efficiency, surpassing traditional implicit neural representations. Ref. [26] combined 3D Gaussians with Neural Implicit Surfaces, jointly optimizing the SDF network. This approach leverages both 3D Gaussians for local geometric details and implicit surface representation for complex geometric structures, though it retains the efficiency limitations of implicit representations. Ref. [8] introduces regularization terms to guide Gaussian distributions toward scene surfaces, combining Gaussian representation with Poisson surface reconstruction for accurate mesh generation. To reduce computational complexity, Ref. [9] proposes a novel approach using 2D Gaussian representations instead of 3D, effectively capturing surface details while improving efficiency. For handling complex geometries, Ref. [27] develops a surface extraction method based on the transcribed signed distance function, particularly effective for reconstructing complex structures and highly reflective objects. Additionally, Ref. [10] presents an innovative approach using tetrahedral decomposition, which transforms rays into local Gaussian coordinate systems for explicit ray-Gaussian intersection computation, significantly enhancing rendering performance in large-scale scenes.

### 2.3. Reconstruction of Reflective Objects

Reconstructing objects with complex reflective properties remains a challenge due to the high surface glossiness and intricate light interactions. Light-field interpolation techniques [28,29,30] enable rendering by combining dense light-field sampling with multiview data collection. Later works explored inverse rendering approaches. For instance, Refs. [31,32] focused on BRDF parameter estimation and reflection model optimization, while [33,34,35] investigated scene lighting estimation and indirect illumination modeling, recovering scene geometry, lighting conditions and material properties from observed images. To specifically address specular reflections, more sophisticated methods [5,36,37] emerged that incorporate advanced reflection models and regularization techniques. However, these improvements [6,38,39] also brought new challenges in computational efficiency. Recent research has focused on balancing reconstruction quality with computational efficiency through several approaches: incorporating specular properties via plane mirror imaging principles [40], by developing efficient lighting formulations for real-time performance [41], and by creating comprehensive frameworks that enable real-time ray tracing through optimized handling of surface normals and BRDF parameters [42]. Unlike Spec-Gaussian [11], which uses ASG primarily for appearance fitting, ARS-GS strictly embeds ASG within a Physically Based Rendering (PBR) pipeline by aligning it with GGX microfacet models. Furthermore, we introduce a novel skip connection for reverse gradient propagation, mitigating vanishing gradients and enhancing geometric fidelity in highly reflective regions.

## 3. Method

**Figure 1:**
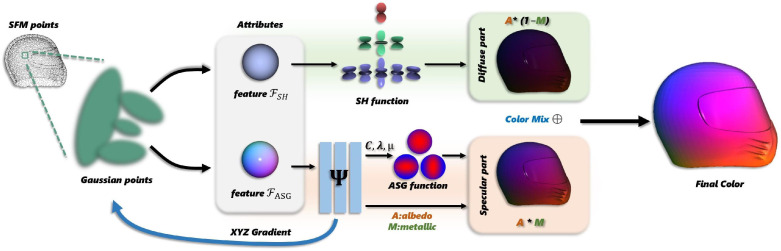
**Overview of our proposed ARS-GS framework.** The pipeline initializes Gaussian primitives with SH features for diffuse appearance and ASG features for reflective characteristics. A skip connection links the ASG network to Gaussian positions, optimizing reconstruction via adaptive Gaussian rasterization. In the diagram, black arrows represent the forward inference process, while blue arrows indicate the backward gradient flow. The asterisk (*) denotes matrix multiplication. The dashed line from SFM points to Gaussian points represents the Gaussian initialization process detailed in Section 3.1. Each square block represents an intermediate variable obtained at the respective stage.

As illustrated in Figure 1, our method progressively reconstructs the information of the scene from multiple images captured in specific poses. The approach places special focus on modeling environmental illumination through a PBR framework, which provides crucial constraints that enable accurate mesh reconstruction through light-transport simulation. Specifically, we initialize Gaussian primitives from a set of images $X_{i}$, where i∈{1,…,N} with known camera poses. Each Gaussian primitive comprises not only fundamental geometric attributes, but also crucial appearance characteristics: color features of spherical harmonics $F_{SH}$ for diffuse characteristics, and material attribute features $F_{ASG}$ for anisotropic Gaussian parameters. Subsequently, these appearance parameters are optimized through a BRDF model, providing physical constraints during training. The final pixel colors are then obtained by integrating the optimized color values with Gaussian opacities through the Gaussian rasterization pipeline (details in Section 3.2). Furthermore, to enhance training efficiency, we implement a shortcut connection mechanism that links the spatial coordinates of each 3D Gaussian primitive directly to its corresponding ASG color attributes. This design enables rapid updates of specular reflections when Gaussian primitives undergo positional changes, as detailed in Section 3.3.

### 3.1. Modeling

We represent the 3D scene as a set of optimizable 3D Gaussian primitives $\left\{G_{k}|k=1,\ldots ,K\right\}$ and render images through Gaussian rasterization. Each 3D Gaussian primitive $G_{k}$ is characterized by its geometric parameters, including opacity $\alpha_{k}\in \left[0,1\right]$, center position $p_{k}\in R^{3\times 1}$, and covariance matrix $\Sigma_{k}\in R^{3\times 3}$. The spatial probability density function of the distribution can be expressed as

(1) $$G_{k}\left(x\right)=\alpha_{k}e^{-\frac{1}{2}{\left(x-p_{k}\right)}^{T}\Sigma_{k}^{-1}\left(x-p_{k}\right)}.$$

Following the approach of Gaussian-opacity fields [10], the final color of a camera ray is computed through explicit modeling of ray-Gaussian intersections and alpha compositing. For each ray r(t)=o+tv, the color is accumulated according to the opacity and transmittance at each intersection point:

(2) $$C\left(o,r\right)=\sum_{j=1}^{J}c_{j}\alpha_{j}E\left(G_{j},o,r\right)\prod_{q=1}^{j-1}\left(1-\alpha_{q}E\left(G_{q},o,r\right)\right),$$

where

(3) $$E\left(G_{j},o,r\right)=G_{j}^{1D}\left(t^{*}\right),$$

where *J* denotes the number of Gaussian primitives contributing along the ray, and $t^{*}$ is the parameter at which the 1D Gaussian distribution $G_{j}^{1D}$ reaches its maximum along the ray r(t), with $G_{j}^{1D}\left(t^{*}\right)$ being the corresponding maximal value. This approach offers the advantage of extracting more accurate mesh models directly through zero-value surface identification, eliminating the need for Poisson reconstruction.

The color $c_{j}$ of each Gaussian can be defined with the PBR equation:

(4) $$c\left(\omega_{o}\right)=\int_{\Omega }L\left(\omega_{i}\right)f\left(\omega_{i},\omega_{o}\right)\left(\omega_{i}\cdot n\right)d\omega_{i},$$

where $\omega_{o}=-v$ describes the view direction, n represents the surface normal vector, and $\omega_{i}$ corresponds to the incoming light direction across the hemisphere Ω. The incident radiance is given by $L\left(\omega_{i}\right)\in {\left[0,+\infty \right)}^{3}$, and the BRDF function [43] is defined as $f\left(\omega_{i},\omega_{o}\right)\in {\left[0,1\right]}^{3}$. The complete BRDF model consists of both diffuse and specular terms and can be propagated as:

(5) $$f\left(\omega_{i},\omega_{o}\right)=\left(1-m\right)\frac{a}{\pi }+\frac{DFG}{4\left(\omega_{i}\cdot n\right)\left(\omega_{o}\cdot n\right)},$$

where m∈[0,1] defines the material’s metallic property, $a\in {\left[0,1\right]}^{3}$ specifies the albedo value. *D*, *F*, and *G* represent the distribution of microfacet normals, the Fresnel reflection coefficient, and the geometric shadowing masking term, respectively. The specific formulations of *D* and *G* have a decisive impact on the modeling of specular reflections.

### 3.2. ASG-Based BRDF Integration

This section focuses on explaining the fundamental principles of the ASG framework and examining its mathematical connections to the microfacet normal distribution *D* and the geometric shadow-masking term *G*. The mathematical representation of the ASG can be formulated as

(6) $$\Phi \left(v;\left[x,y,z\right],\left[\lambda ,\mu \right],A\right)=A\cdot S\left(v;z\right)\cdot e^{-\lambda {\left(v\cdot x\right)}^{2}-\mu {\left(v\cdot y\right)}^{2}},$$

where z, x, and y are the lobe, tangent, and bitangent axes of the surface, respectively. [x,y,z] forms an orthonormal frame. λ and μ are the bandwidths for the x- and y-axes, respectively, satisfying λ,μ>0. The parameter *A* represents the lobe amplitude. S(v;z)=max(v·z,0) represents the attenuation term based on the view direction. The exponential term encapsulates the anisotropic decay based on the view direction v.

#### 3.2.1. Handling the Distribution Term $D_{ASG}$

**Figure 2:**
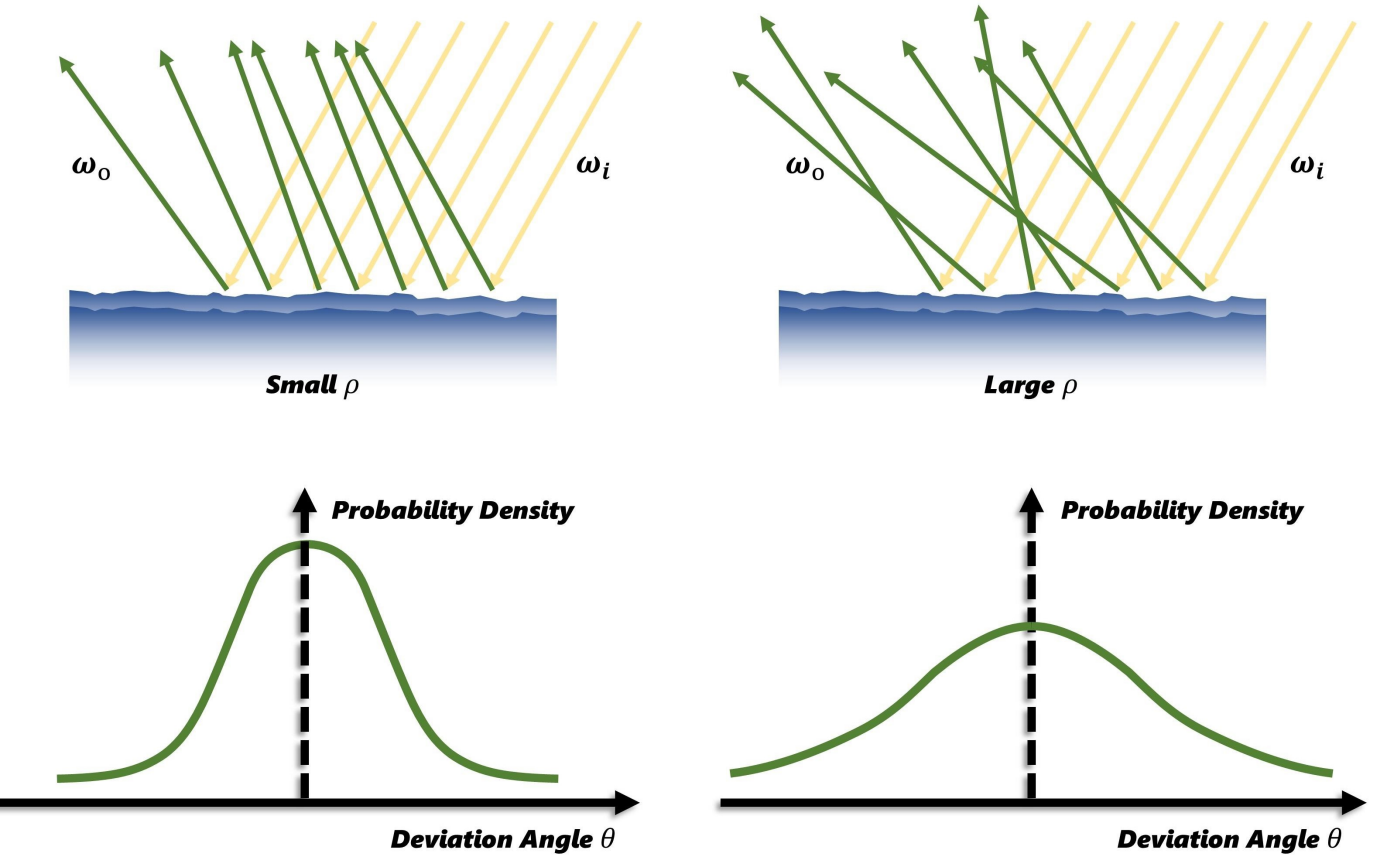
Visualization of GGX microfacet distribution function. The plots illustrate the variation in the distribution term *D* with different roughness parameters ρ. In the figure, the yellow arrows represent the incident light direction $\omega_{i}$, and the green arrows represent the outgoing light direction $\omega_{o}$.

In BRDF model, the GGX microfacet distribution function [44] is commonly used to describe the distribution term *D* as

(7) $$D\left(h\right)=\frac{\rho^{2}}{\pi {\left[{\left(n\cdot h\right)}^{2}\left(\rho^{2}-1\right)+1\right]}^{2}},$$

where ρ denotes the roughness parameter. As illustrated in Figure 2, the higher values indicate rougher surfaces. $h=\frac{\omega_{o}+\omega_{i}}{∥\omega_{o}+\omega_{i}∥}$ is the half-way vector.

To align (7) with the ASG framework, we can consider a local surface coordinate system [x,y,z] on the Gaussian primitive, where z is the surface normal. For smooth surfaces, where the half-vector h predominantly aligns with the z direction, we employ a second-order Taylor expansion:

(8) $$h\cdot z\approx 1-\frac{1}{2}\left[{\left(h\cdot x\right)}^{2}+{\left(h\cdot y\right)}^{2}\right].$$

Substituting (8) into the GGX distribution and neglecting higher-order terms in the Taylor expansion, the denominator can be well-approximated by an ASG lobe with matched anisotropic bandwidths:

(9) $$D_{ASG}\left(h\right)\approx \frac{\lambda \mu }{\pi {\left[\lambda {\left(h\cdot x\right)}^{2}+\mu {\left(h\cdot y\right)}^{2}\right]}^{2}}\approx \Phi \left(h;\left[x,y,z\right],\left[\lambda ,\mu \right]\right),$$

with the small-angle approximation and after matching the lobe width, which is a practical approximation rather than an exact equivalence:

(10) $$\lambda =\mu =\frac{1-\rho^{2}}{\rho^{2}}.$$

**Figure 3:**
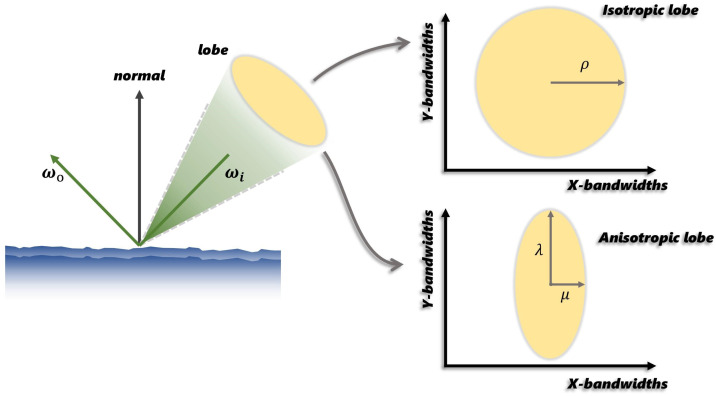
**Comparison between isotropic and anisotropic distribution lobes.** **Left**: Light interaction with a rough surface. **Right**: Isotropic distribution (top, controlled by ρ) versus anisotropic distribution (bottom, controlled by λ and μ).

As illustrated in Figure 3, ASG provides anisotropic control along the x and y axes through parameters λ and μ, which govern the distribution spread and the anisotropic roughness in the BRDF, ensuring consistency between the distribution and geometric terms.

#### 3.2.2. Handling the Geometry Term $G_{ASG}$

Beyond modeling anisotropic microfacet distributions, the geometry term $G_{ASG}$ captures the shadowing and masking effects between microfacets, enhancing the physical accuracy of specular reflections.

Schlick–GGX formulations typically depend on the angles between the view and light directions relative to the surface normal as

(11) $$G\left(\omega_{i},\omega_{o}\right)=G_{1}\left(\omega_{i}\right)\cdot G_{1}\left(\omega_{o}\right),$$

Following a similar derivation process, we can obtain the masking function $G_{1}\left(\omega \right)$ for a single direction and reparameterize the geometry term to share anisotropy with the distribution via the same λ and μ, which is an approximation aligning masking with anisotropic roughness for consistency with the anisotropic lobe:

(12) $$G_{1}\left(\omega \right)=\frac{2(n\cdot \omega )}{\left(n\cdot \omega \right)+\sqrt{\lambda {\left(h\cdot x\right)}^{2}+\mu {\left(h\cdot y\right)}^{2}+{\left(n\cdot \omega \right)}^{2}}},$$

Here, λ and μ are the ASG bandwidths controlling the anisotropic distribution and simultaneously dictate the roughness-related scaling in the geometry term.

#### 3.2.3. Handling the Specular Component

Combining $D_{ASG}$ with $G_{ASG}$ and the Fresnel term *F* yields a comprehensive specular component within the BRDF:

(13) $$f{\left(\omega_{i},\omega_{o}\right)}_{ASG}=\frac{D_{ASG}\cdot F\cdot G_{ASG}}{4\left(\omega_{i}\cdot n\right)\left(\omega_{o}\cdot n\right)}.$$

The specular reflectance of each Gaussian primitive along the ray is formulated with an anisotropic geometric attenuation term as

(14) $$c_{\mathrm{specular},j}=\int_{\Omega }\frac{D_{ASG}\cdot F\cdot G_{ASG}}{4\left(\omega_{i}\cdot n\right)\left(\omega_{o}\cdot n\right)}L\left(\omega_{i}\right)\left(\omega_{i}\cdot n\right)\;d\omega_{i},$$

where $c_{\mathrm{specular},j}$ represents the specular color of the *j*-th Gaussian primitive. To address the computational complexity of (14), we implement the split-sum approximation introduced in [45]. This approximation decomposes the integral into two separable terms:

(15) $$c_{\mathrm{specular},j}\approx \underset{\mathrm{Lighting}\;\mathrm{integral}}{\underset{︸}{\int_{\Omega }L\left(\omega_{i}\right)D_{ASG}\left(\rho ,t\right)d\omega_{i}}}\cdot \underset{\mathrm{BRDF}\;\mathrm{integral}}{\underset{︸}{\int_{\Omega }\frac{D_{ASG}FG_{ASG}}{4(\omega_{o}\cdot n)}d\omega_{i}}}$$

The first term represents the integration of incident radiance weighted by $D_{ASG}\left(\rho ,t\right)\in \left[0,1\right]$ (characterized as the specular lobe) as depicted in Figure 3, where t denotes the reflective direction. The second term covers the BRDF integral. The first light integral term can be efficiently calculated with the following equation as proven in [5]:

(16) $$\int_{\Omega }L_{SH}\left(\omega_{i}\right)D_{ASG}\left(\rho ,t\right)\;d\omega_{i}=\sum_{\ell ,m}A_{\ell }\left(\frac{1}{\rho }\right)c_{\ell m}Y_{\ell }^{m}\left(t\right),$$

where $L_{SH}\left(\omega_{i}\right)$ represents the incident light direction encoded using spherical harmonics. $A_{\ell }\left(\frac{1}{\rho }\right)\approx \exp\left(-\frac{2}{\rho \ell (\ell +1)}\right)$ is the attenuation factor. $c_{\ell m}$ represents the spherical harmonic expansion coefficients. $Y_{\ell }^{m}\left(t\right)$ denotes the spherical harmonic basis function evaluated at the reflection direction t. The result of the integral then serves as the input to an MLP to predict the integrated light intensity. According to [6], the BRDF integral can be efficiently computed as $\left(\left(1-m\right)\cdot 0.04+m\cdot a\right)\cdot F_{1}+F_{2}$, where $F_{1}$ and $F_{2}$ are precomputed lookup tables parameterized by roughness.

#### 3.2.4. Handling the Diffuse Component

The diffuse component of each Gaussian primitive depends on the dot product between the incident light direction and the surface normal. We utilize existing spherical harmonics to fit the diffuse light, keeping the formula for the diffuse component unchanged:

(17) $$c_{\mathrm{diffuse},j}=a_{j}\left(1-m_{j}\right)\underset{L_{\mathrm{diffuse}}}{\underset{︸}{\int_{\Omega }L_{SH}\left(\omega_{i}\right)\frac{\omega_{i}\cdot n}{\pi }d\omega_{i}}}.$$

The cosine lobe $\frac{\omega_{i}\cdot n}{\pi }$ serves as the clamped-cosine kernel for irradiance, and the integral is efficiently computed via SH convolution. Following an analogous methodology to (16), we can compute the integral for the diffuse component of the reflection model.

#### 3.2.5. Final Color Calculation

Per-primitive shaded colors are obtained by summing the diffuse and specular components for each Gaussian primitive:

(18) $$c_{j}=c_{\mathrm{diffuse},j}+c_{\mathrm{specular},j},$$

and the final pixel color is then produced by alpha compositing these per-primitive colors along the ray according to (2).

**Figure 4:**
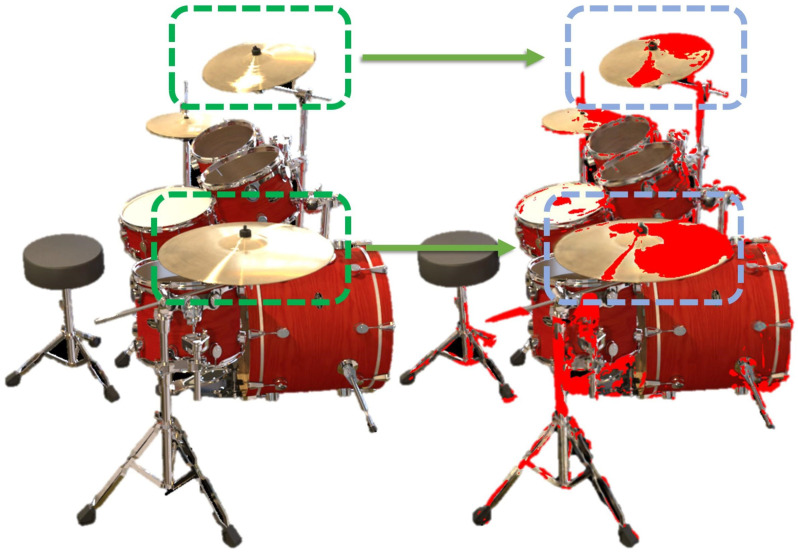
Analysis of Specular Reflection Modeling. Specular regions (marked in red) highlight where our physically grounded approach precisely models light-surface interactions, accurately capturing view-dependent effects across diverse geometries. In addition, the green dashed boxes indicate the specular highlights in the ground truth (original) images, while the blue dashed boxes represent the specular highlights calculated by our proposed algorithm. As shown, our method can accurately fit the specular highlights in real images.

As shown in Figure 4, red regions indicate pixels where ASG components contribute over 70% of the total reflectance intensity (from the grayscale BRDF output). These areas exhibit strong, view-dependent specularities that spherical-harmonics bases alone cannot capture, highlighting the effectiveness of combining $D_{\mathrm{ASG}}$ with $G_{\mathrm{ASG}}$ for efficient and realistic rendering.

### 3.3. Reverse Gradient Propagation Optimization

For scenes with dense specular reflections, the traditional gradient path from reflectance color loss to Gaussian positions can lead to vanishing gradients. We propose a skip connection mechanism to directly link specular reflectance with Gaussian positions.

Conventionally, the gradients in world coordinates for each Gaussian primitive follow:

(19) $$\frac{\partial L}{\partial p_{w}}=\frac{\partial L}{\partial c_{i}}\left(\frac{\partial c_{i}}{c_{\mathrm{diffuse}}}\frac{c_{\mathrm{diffuse}}}{\partial v_{g}}+\frac{\partial c_{i}}{c_{\mathrm{specular}}}\frac{c_{\mathrm{specular}}}{\partial v_{g}}\right)\frac{\partial v_{g}}{\partial p_{w}},$$

where the viewing direction $v_{g}\in R^{3}$ is defined as:

(20) $$v_{g}=\frac{p_{w}-p_{c}}{|p_{w}-p_{c}{|}_{2}}.$$

Here, $p_{w}$ and $p_{c}\in R^{3}$ denote the Gaussian and camera center positions in world coordinates, respectively.

To address the gradient limitation, we design an ASG decoder network incorporating both material properties and spatial information. This enables direct gradient propagation:

(21) $$\frac{\partial L}{\partial p_{w}}=\frac{\partial L}{\partial c_{\mathrm{specular}}}\frac{\partial c_{\mathrm{specular}}}{\partial p_{w}},$$

This optimization accelerates convergence in specular regions and improves mesh extraction completeness, demonstrating superior performance in both accuracy and efficiency.

## 4. Experiment

In this section, we compare ARS-GS with existing methods in multiple datasets. Specifically, we selected the Ref-NeRF real-world dataset [5] for its complex real-world specular reflections and directional lighting, the NeRF-Synthetic dataset [1] for its geometrically intricate objects with varying material properties, and the Shiny Blender dataset [46] to rigorously test performance on highly glossy and challenging synthetic metallic surfaces. To evaluate the effectiveness of our method, we evaluated the quality of the rendered RGB image and reconstruction of the mesh containing highly specular scenes. Furthermore, we selected several common scenes from the Mip-NeRF-360 dataset [13], which features unbounded outdoor and indoor environments with complex backgrounds, to validate the expressiveness of our algorithm in conventional scenarios. For all scenes, we present both RGB images and normal maps to better demonstrate the geometric reconstruction quality. Ablation studies verify the effectiveness of each component of our algorithm.

### 4.1. Implementation Details

The proposed method is developed using PyTorch (version 2.0.1), where we enhanced the original Gaussian rasterization pipeline by incorporating ASG rendering capabilities. The disentanglement network of ASG features Ψ consists of a three-layer configuration, employing 64-dimensional hidden representations per layer, with a skip connection applied at the second layer to preserve high-frequency details. For directional encoding, we use second-degree spherical harmonics or Fourier features on the view direction. All experiments are performed on an NVIDIA A100 GPU. The model is trained for 30,000 iterations using the Adam optimizer. The learning rate for the MLP network is set to $1\times 10^{-3}$, while the learning rates for the 3D Gaussian parameters follow the standard 3DGS schedule, with the position learning rate decaying exponentially from $1.6\times 10^{-4}$ to $1.6\times 10^{-6}$. The volumetric parameters are empirically selected, with an initial voxel resolution ϵ of $1\times 10^{-3}$ and an expansion coefficient β set to 4.Furthermore, to evaluate computational efficiency, we measured the training time and inference speed of our approach. On average, our method requires approximately 45 min for training per scene. During inference, it achieves a real-time rendering speed of 85 frames per second (FPS) at a resolution of 1920×1080, demonstrating that the incorporation of ASG and physical illumination models maintains high computational efficiency without significant overhead.

### 4.2. Results and Comparisons

#### 4.2.1. Results on Specular Scenes

We evaluate our algorithm against state-of-the-art methods on three datasets: Ref-NeRF (real-world), NeRF (synthetic), and Shiny Blender. The comparison includes neural radiance field methods (Ref-NeRF, Mip-NeRF) and 3D Gaussian approaches (3DGS, GOF, Spec-Gaussian). Performance is assessed using PSNR, SSIM, and LPIPS for methodological consistency. Specifically, PSNR measures pixel-level reconstruction accuracy, SSIM evaluates structural similarity and texture preservation, and LPIPS assesses perceptual image quality based on deep feature representations. For scenes not previously evaluated in prior work, we implement the baselines from their official repositories, adhering to default hyperparameters and training protocols.

As shown in Table 1, our method achieves superior performance across multiple metrics. Specifically, it attains the highest PSNR scores on Sedan (26.26) and Toy-car (23.88) scenes, while achieving the best SSIM (0.589) on Gardensphere and the lowest LPIPS (0.246) scores.

**Figure 5:**
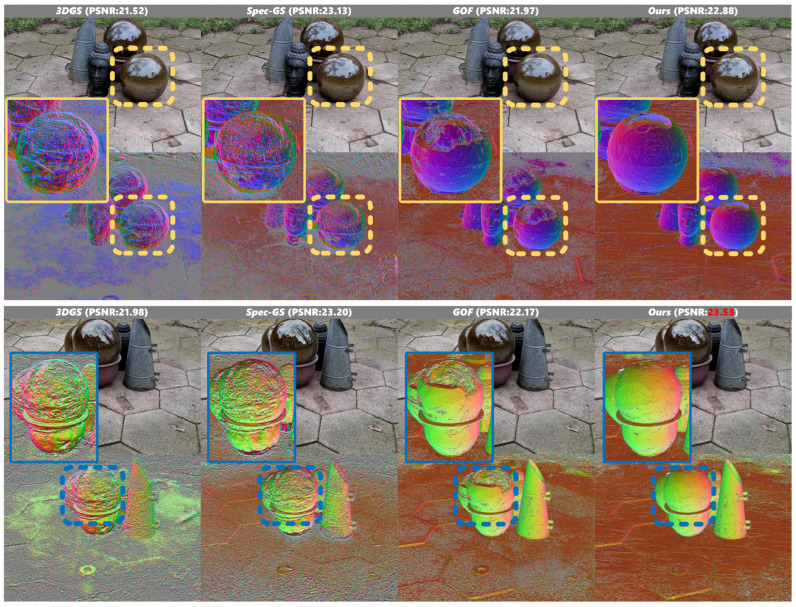
Qualitative results on Ref-NeRF dataset scene 1. In the figure, the dashed boxes indicate specific regions of interest in the original images, while the corresponding solid boxes of the same color (yellow and blue) display the magnified local views of these regions for detailed comparison.

Detailed examination of the magnified views in Figure 5, particularly in the garden sphere scene from the Ref-NeRF dataset, reveals a significant advantage of our approach in handling challenging specular surfaces. When confronted with highly reflective metallic spheres characterized by rapid variations in specular reflections, existing methods struggle to generate complete geometric reconstructions, exhibiting substantial mesh deterioration in regions of high specularity. In contrast, our algorithm successfully reconstructs the complete spherical geometry while preserving the intricate reflective properties.

**Figure 6:**
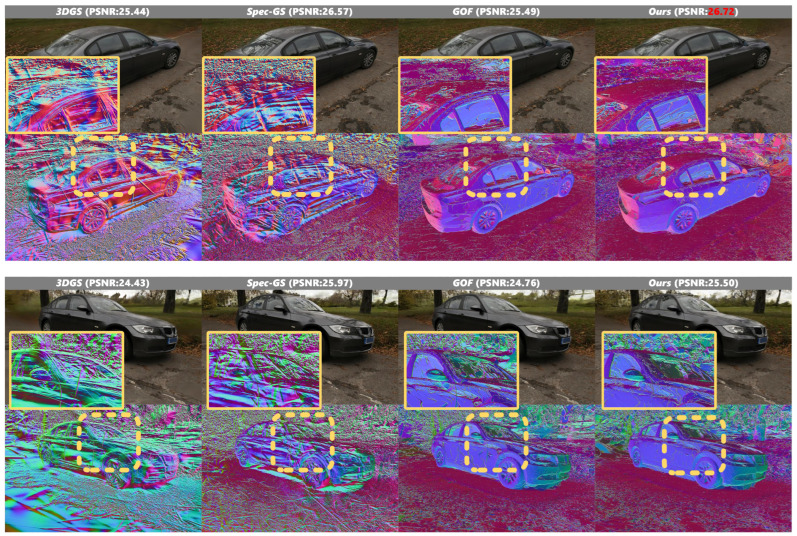
Qualitative results on Ref-NeRF dataset scene 2. In the figure, the dashed boxes indicate specific regions of interest in the original images, while the corresponding solid boxes of the same color (yellow and blue) display the magnified local views of these regions for detailed comparison.

The automotive scene in Figure 6 presents additional challenges due to its geometric complexity and suboptimal illumination conditions. In the real-world dataset, we encounter an inherently under-constrained optimization problem where, lacking precise surface information and light occlusion conditions, multiple possible solutions exist for light transport constraints when solely relying on multiview image information. Although our reconstruction results still leave room for improvement, the optimization trajectory nonetheless demonstrates convergence toward physically plausible solutions, suggesting the effectiveness of our approach in handling such ambiguous scenarios.

**Figure 7:**
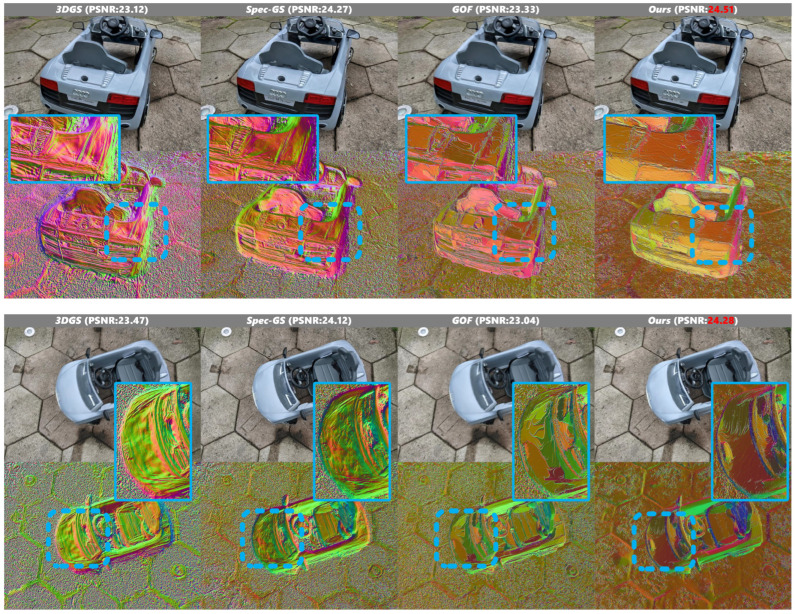
Qualitative results on Ref-NeRF dataset scene 3. In the figure, the dashed boxes indicate specific regions of interest in the original images, while the corresponding solid boxes of the same color (yellow and blue) display the magnified local views of these regions for detailed comparison.

Furthermore, in the toy car scene illustrated in Figure 7, our method faithfully reconstructs the specular surfaces of the vehicle, accurately capturing the smooth reflective properties and generating the most geometrically accurate mesh representation among all compared methods.

Our method demonstrates superior performance on both NeRF-Synthetic and Gloss-Blender dataset as shown in Table 2 and Table 3. On NeRF-Synthetic, we achieve leading scores in PSNR (up to 38.30), SSIM (up to 0.997), and LPIPS (as low as 0.011). Similar advantages are observed on Gloss-Blender, particularly in handling glossy surfaces, with PSNR reaching 46.31 on the Teapot scene.

**Figure 8:**
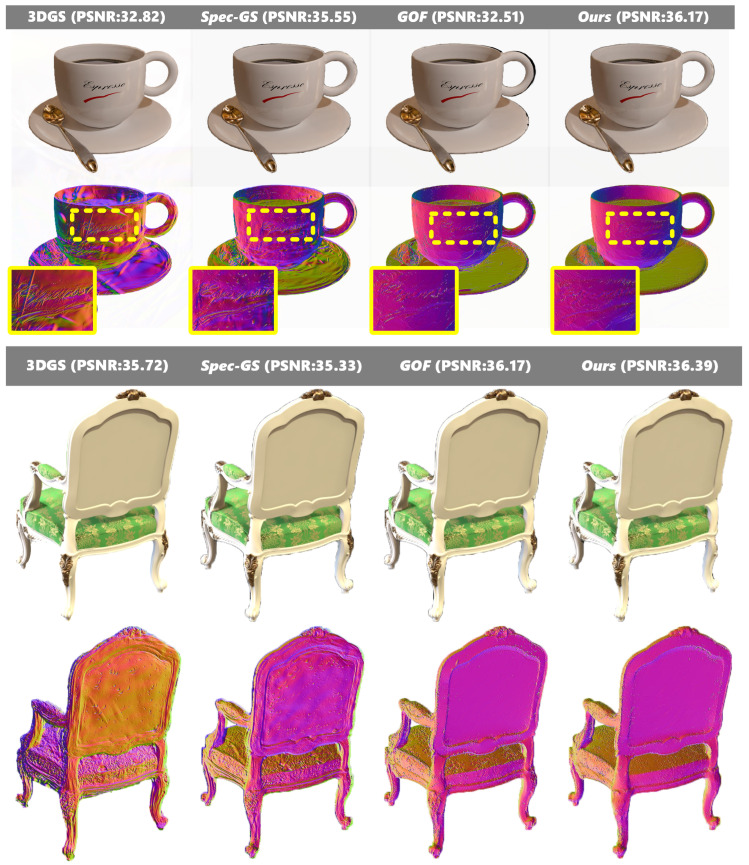
Qualitative results on NeRF-Synthetic dataset and Gloss-Blender dataset.In the figure, the dashed boxes indicate specific regions of interest in the original images, while the corresponding solid boxes of the same color (yellow and blue) display the magnified local views of these regions for detailed comparison.

Our method demonstrates superior performance across synthetic scenarios Figure 8. In the coffee scene from NeRF-Synthetic dataset, our approach excels in handling planar surface details. While existing methods produce geometric distortions on the coffee cup’s smooth surface due to unconstrained illumination equations, our method successfully preserves surface smoothness. Similarly, in the chair example from Gloss-Blender dataset, our approach accurately captures the flat geometry of the chair’s backrest with high fidelity.

#### 4.2.2. Results on General Scenes

**Figure 9:**
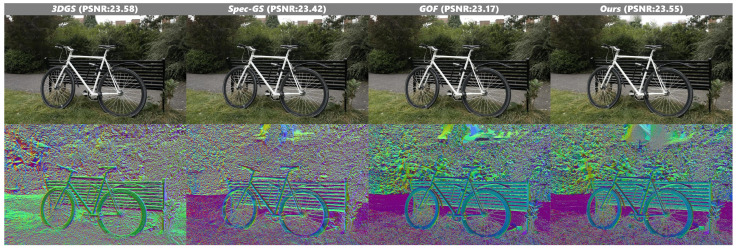
Qualitative results on Mip-NeRF dataset bicycle. In the figure, the dashed boxes indicate specific regions of interest in the original images, while the corresponding solid boxes of the same color (yellow and blue) display the magnified local views of these regions for detailed comparison.

Our method achieves competitive results across all Mip-NeRF benchmark scenes (Table 4). Notably, we obtain the highest PSNR (32.20) on the Kitchen scene and superior SSIM scores (0.892, 0.780) for Garden and Bicycle scenes. The consistently low LPIPS values (0.108, 0.195, 0.119) further demonstrate our method’s effectiveness in preserving perceptual quality. As demonstrated in Figure 9, our method can accurately reconstruct geometry in general scenes.

### 4.3. Computational Complexity and Execution Time

To comprehensively evaluate the efficiency of our proposed ARS-GS framework, we conducted a comparative analysis of computational complexity, execution time, and memory consumption against baseline methods. The evaluations were performed on an NVIDIA A100 GPU using the NeRF-Synthetic dataset at a resolution of 800×800. As presented in Table 5, our method achieves an inference speed of 85 FPS, which maintains real-time rendering capabilities comparable to the original 3DGS and GOF, while significantly outperforming NeRF-based methods. The training time for ARS-GS averages approximately 45 min per scene. The introduction of the ASG network and physically based rendering constraints introduces a moderate increase in training time (around 15–20% compared to standard 3DGS) and a slight increase in VRAM usage (6.2 GB). However, this computational trade-off is well justified by the substantial improvements in rendering quality and geometric fidelity for highly reflective surfaces, demonstrating that ARS-GS successfully balances physical accuracy with computational efficiency.

### 4.4. Ablation Studies

To quantitatively evaluate the contribution of each component in our proposed ARS-GS framework, we conduct ablation studies on the Ref-NeRF dataset. The numerical results are summarized in Table 6.

#### 4.4.1. ASG and BRDF Color Structure

**Figure 10:**
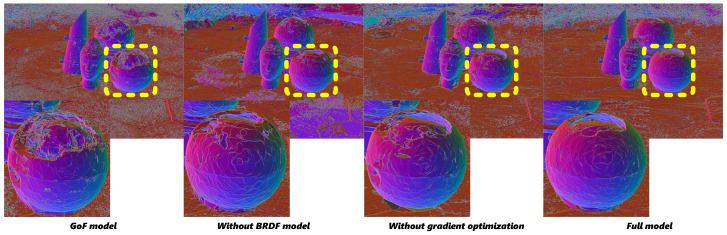
Ablation on PBR model and gradient optimization. Our proposed optimization strategy effectively addresses the surface quality degradation and inconsistent specular reflections caused by directly employing the PBR model without gradient optimization. In the figure, the dashed boxes indicate specific regions of interest in the original images, while the corresponding solid boxes of the same color (yellow and blue) display the magnified local views of these regions for detailed comparison.

We conducted ablation studies to evaluate the effectiveness of using physical illumination models for reconstruction, with particular emphasis on challenging scenarios involving highly reflective surfaces. Our analysis demonstrates that traditional reconstruction methods without PBR modeling often fail to accurately capture and reconstruct mesh structures in regions with strong specular properties. The experiments reveal significant mesh incompleteness and geometric artifacts in these reflective areas when physical illumination constraints are not incorporated, as shown in Figure 10. These results validate that our physically grounded approach is essential for complete and accurate mesh reconstruction in scenes containing challenging reflective elements, where conventional techniques typically exhibit significant reconstruction deficiencies.

#### 4.4.2. Gradient Optimization Strategy

We performed ablation studies to evaluate the effectiveness of our gradient optimization approach. Our experiments demonstrate that direct application of the PBR model without the gradient optimization strategy results in suboptimal reconstruction quality and incomplete mesh generation. Compared to implementations without our gradient optimization, we observe substantially more complete mesh reconstruction, particularly in geometrically complex regions. The shorter gradient path enables more precise control over Gaussian primitive optimization, resulting in better-preserved surface details and a more coherent overall structure. This improvement is particularly evident in areas where traditional approaches struggle to maintain the continuity of reconstruction.

### 4.5. Discussion

In this section, we revisit the research questions (RQs) formulated in the introduction and validate them based on our experimental findings.

#### 4.5.1. Validation of RQ1

The integration of ASG and SH within a PBR framework is proven highly effective for modeling complex reflections. As demonstrated in our ablation studies (Table 6 and Figure 10), the removal of the PBR model leads to a significant drop in rendering quality (e.g., average PSNR drops from 24.02 to 22.45 on the Ref-NeRF dataset) and severe geometric artifacts in reflective regions. This confirms that our ASG-based BRDF integration successfully captures the physical constraints of light transport, thereby answering RQ1 affirmatively.

#### 4.5.2. Validation of RQ2

The proposed skip connection architecture directly links the ASG specular reflectance with Gaussian spatial coordinates, which mitigates the vanishing gradient problem in highly specular regions. Our ablation study on the gradient optimization strategy (Table 6 and Figure 10) shows that without this strategy, the mesh reconstruction is incomplete and the PSNR decreases to 23.80. The full model preserves surface details and maintains structural coherence, directly validating RQ2.

#### 4.5.3. Validation of RQ3

The comprehensive evaluations across the Ref-NeRF, NeRF-Synthetic, Gloss-Blender, and Mip-NeRF datasets validate the superiority of our framework. ARS-GS consistently outperforms state-of-the-art methods (such as 3DGS, GOF, and Spec-Gaussian) in PSNR, SSIM, and LPIPS metrics, particularly in scenes with high specularity (e.g., achieving a PSNR of 46.31 on the Gloss-Blender Teapot scene and 26.26 on the Ref-NeRF Sedan scene). These quantitative and qualitative results provide a robust affirmative answer to RQ3, demonstrating the framework’s exceptional rendering quality and geometric fidelity.

## 5. Conclusions

We introduce ARS-GS, an algorithm integrating ASG with BRDF modeling for 3D reconstruction. Built on the 3DGS framework, our approach explicitly models diffuse and specular components, significantly improving reconstruction accuracy in specular environments. Experimental results demonstrate ARS-GS’s superior performance over existing 3DGS-based methods in both surface reconstruction and novel view synthesis tasks.

Despite these promising outcomes, our method has certain limitations. First, the incorporation of physically based rendering constraints and the ASG network introduces additional computational overhead during the training phase relative to the baseline 3DGS framework. Second, the current BRDF integration is predicated on simplified light transport assumptions, which may be inadequate for accurately modeling highly complex global illumination phenomena, such as higher-order (multi-bounce) reflections and pronounced inter-reflections. Future work will therefore focus on (i) computational optimizations to reduce training time, (ii) the integration of more sophisticated physically based light transport models to better capture complex global illumination effects, and (iii) extending the proposed framework to dynamic scenes and real-time interactive applications. 

## Figures and Tables

**Table 1:** Quantitative evaluation on the Ref-NeRF dataset. The best and second best results are highlighted.

	PSNR			SSIM			LPIPS		
Method	Gardensphere	Sedan	Toy-Car	Gardensphere	Sedan	Toy-Car	Gardensphere	Sedan	Toy-Car
Ref NeRF	22.01	25.21	23.65	0.584	0.720	0.633	0.251	0.234	0.231
3DGS	21.75	26.03	23.78	0.571	0.711	0.637	0.248	0.206	0.237
GOF	21.56	25.99	23.69	0.562	0.701	0.642	0.252	0.203	0.249
ASG	21.90	26.10	23.82	0.572	0.708	0.650	0.258	0.208	0.241
Ours	21.93	26.26	23.88	0.589	0.713	0.648	0.246	0.204	0.233

**Table 2:** Quantitative results on NeRF-Synthetic dataset. The best and second best results are highlighted.

Metric	Method	Chair	Drums	Ficus	Hotdog	Lego	Materials	Mic	Ship
PSNR	Mip-NeRF	32.89	25.58	31.80	35.40	32.24	29.46	33.26	29.88
	3DGS	35.36	26.15	34.87	37.72	35.78	30.00	30.82	30.80
	GOF	32.37	26.44	34.29	35.06	34.88	29.88	31.34	30.95
	Spec-GS	35.68	26.92	36.14	38.28	36.07	30.85	37.12	31.89
	Ours	35.53	26.94	36.18	38.30	35.73	30.88	37.21	31.85
SSIM	Mip-NeRF	0.974	0.939	0.981	0.982	0.973	0.969	0.987	0.915
	3DGS	0.915	0.851	0.921	0.930	0.882	0.882	0.909	0.827
	GOF	0.922	0.873	0.911	0.955	0.884	0.897	0.899	0.840
	Spec-GS	0.987	0.958	0.988	0.985	0.982	0.963	0.993	0.909
	Ours	0.979	0.965	0.981	0.990	0.988	0.965	0.997	0.920
LPIPS	Mip-NeRF	0.033	0.062	0.022	0.025	0.030	0.041	0.023	0.138
	3DGS	0.047	0.087	0.055	0.034	0.064	0.055	0.046	0.113
	GOF	0.012	0.040	0.013	0.027	0.057	0.050	0.037	0.094
	Spec-GS	0.011	0.032	0.011	0.019	0.014	0.032	0.006	0.104
	Ours	0.011	0.028	0.012	0.017	0.012	0.030	0.008	0.100

**Table 3:** Quantitative results on Gloss-Blender dataset. The best and second best results are highlighted.

Metric	Method	Ball	Car	Coffee	Helmet	Teapot	Toaster
PSNR	Ref-NeRF	33.16	30.44	33.99	29.94	45.12	26.12
	3DGS	27.65	27.26	32.30	28.22	45.71	20.99
	GOF	28.46	27.58	31.89	28.31	44.98	21.56
	Spec-GS	34.13	32.11	35.16	30.85	46.09	26.04
	Ours	34.50	31.98	35.20	30.98	46.31	25.89
SSIM	Ref-NeRF	0.956	0.949	0.972	0.955	0.995	0.910
	3DGS	0.937	0.930	0.971	0.951	0.996	0.895
	GOF	0.925	0.933	0.965	0.954	0.992	0.887
	Spec-GS	0.964	0.953	0.977	0.960	0.996	0.912
	Ours	0.958	0.955	0.982	0.963	0.995	0.910
LPIPS	Ref-NeRF	0.307	0.051	0.082	0.087	0.013	0.118
	3DGS	0.161	0.047	0.078	0.079	0.007	0.126
	GOF	0.177	0.052	0.088	0.080	0.015	0.125
	Spec-GS	0.155	0.043	0.074	0.073	0.007	0.122
	Ours	0.152	0.042	0.077	0.070	0.008	0.121

**Table 4:** Quantitative results on Mip-NeRF dataset. The best and second best results are highlighted.

	PSNR			SSIM			LPIPS		
Method	Garden	Bicycle	Kitchen	Garden	Bicycle	Kitchen	Garden	Bicycle	Kitchen
Mip-NeRF	23.16	21.69	26.47	0.543	0.454	0.745	0.422	0.541	0.336
3DGS	27.41	25.25	31.44	0.868	0.771	0.922	0.103	0.205	0.129
GOF	26.18	24.35	28.11	0.860	0.650	0.740	0.157	0.205	0.147
ASG	27.50	25.12	32.10	0.880	0.775	0.919	0.114	0.197	0.128
Ours	27.41	25.22	32.20	0.892	0.780	0.919	0.108	0.195	0.119

**Table 5:** Comparison of computational complexity, training time, and inference speed (averaged across the NeRF-Synthetic dataset). The arrows ↓ and ↑ indicate that lower and higher values are better, respectively. The best results and our proposed method are highlighted in bold.

Method	Training Time (min) ↓	Inference Speed (FPS) ↑	VRAM (GB) ↓
Mip-NeRF	∼600	<0.1	14.5
3DGS	**35**	**120**	**4.5**
GOF	42	95	5.1
Spec-GS	48	75	6.8
**Ours (ARS-GS)**	45	85	6.7

**Table 6:** Quantitative ablation study results (average over Ref-NeRF scenes). We evaluate the impact of the PBR model and the gradient optimization strategy. The arrows ↑ and ↓ indicate that higher and lower values are better, respectively. The best results and our full model are highlighted in bold.

Model Variations	PSNR ↑	SSIM ↑	LPIPS ↓
w/o PBR	22.45	0.612	0.285
w/o Grad. Opt.	23.80	0.635	0.258
**Full Model (Ours)**	**24.02**	**0.650**	**0.227**

## Data Availability

The raw data and code supporting the conclusions of this article will be made available by the authors on request.

## Abbreviations

The following abbreviations are used in this manuscript:

3DGS	3D Gaussian Splatting
ARS-GS	Anisotropic Reflective Spherical 3D Gaussian Splatting
ASG	Anisotropic Spherical Gaussian
BRDF	Bidirectional Reflectance Distribution Function
FPS	Frames Per Second
GOF	Gaussian-Opacity Fields
LPIPS	Learned Perceptual Image Patch Similarity
MLP	Multi-Layer Perceptron
NeRF	Neural Radiance Fields
NVS	Novel View Synthesis
PBR	Physically Based Rendering
PSNR	Peak Signal-to-Noise Ratio
SDF	Signed Distance Field
SH	Spherical Harmonics
SLAM	Simultaneous Localization and Mapping
SSIM	Structural Similarity Index Measure
VR	Virtual Reality

## References

[1] Mildenhall B., Srinivasan P.P., Tancik M., Barron J.T., Ramamoorthi R., Ng R. (2021). NeRF: Representing scenes as neural radiance fields for view synthesis. Commun. ACM.

[2] Wang P., Liu L., Liu Y., Theobalt C., Komura T., Wang W. (2021). NeuS: Learning Neural Implicit Surfaces by Volume Rendering for Multi-view Reconstruction. Proceedings of the Advances in Neural Information Processing Systems.

[3] Yang X., Li H., Zhai H., Ming Y., Liu Y., Zhang G. (2022). Vox-fusion: Dense tracking and mapping with voxel-based neural implicit representation. 2022 IEEE International Symposium on Mixed and Augmented Reality (ISMAR).

[4] Yu Z., Peng S., Niemeyer M., Sattler T., Geiger A. (2022). MonoSDF: Exploring monocular geometric cues for neural implicit surface reconstruction. Adv. Neural Inf. Process. Syst..

[5] Verbin D., Hedman P., Mildenhall B., Zickler T., Barron J.T., Srinivasan P.P. (2022). Ref-NeRF: Structured view-dependent appearance for neural radiance fields. 2022 IEEE/CVF Conference on Computer Vision and Pattern Recognition (CVPR).

[6] Liu Y., Wang P., Lin C., Long X., Wang J., Liu L., Komura T., Wang W. (2023). NeRO: Neural geometry and BRDF reconstruction of reflective objects from multiview images. ACM Trans. Graph. (TOG).

[7] Kerbl B., Kopanas G., Leimkühler T., Drettakis G. (2023). 3D Gaussian Splatting for Real-Time Radiance Field Rendering. ACM Trans. Graph. (TOG).

[8] Guédon A., Lepetit V. SuGaR: Surface-aligned gaussian splatting for efficient 3D mesh reconstruction and high-quality mesh rendering. Proceedings of the IEEE/CVF Conference on Computer Vision and Pattern Recognition.

[9] Huang B., Yu Z., Chen A., Geiger A., Gao S. (2024). 2D Gaussian Splatting for geometrically accurate radiance fields. Proceedings of the ACM SIGGRAPH 2024 Conference Papers.

[10] Yu Z., Sattler T., Geiger A. (2024). Gaussian opacity fields: Efficient adaptive surface reconstruction in unbounded scenes. ACM Trans. Graph. (TOG).

[11] Yang Z., Gao X., Sun Y., Huang Y., Lyu X., Zhou W., Jiao S., Qi X., Jin X. (2024). Spec-Gaussian: Anisotropic View-Dependent Appearance for 3D Gaussian Splatting. Proceedings of the Advances in Neural Information Processing Systems.

[12] Xu K., Sun W.L., Dong Z., Zhao D.Y., Wu R.D., Hu S.M. (2013). Anisotropic spherical gaussians. ACM Trans. Graph. (TOG).

[13] Barron J.T., Mildenhall B., Tancik M., Hedman P., Martin-Brualla R., Srinivasan P.P. Mip-NeRF: A multiscale representation for anti-aliasing neural radiance fields. Proceedings of the IEEE/CVF International Conference on Computer Vision.

[14] Barron J.T., Mildenhall B., Verbin D., Srinivasan P.P., Hedman P. Mip-NeRF 360: Unbounded anti-aliased neural radiance fields. Proceedings of the IEEE/CVF Conference on Computer Vision and Pattern Recognition.

[15] Guo H., Peng S., Lin H., Wang Q., Zhang G., Bao H., Zhou X. Neural 3D scene reconstruction with the manhattan-world assumption. Proceedings of the IEEE/CVF Conference on Computer Vision and Pattern Recognition.

[16] Oechsle M., Peng S., Geiger A. UNISURF: Unifying neural implicit surfaces and radiance fields for multi-view reconstruction. Proceedings of the IEEE/CVF International Conference on Computer Vision.

[17] Li Z., Müller T., Evans A., Taylor R.H., Unberath M., Liu M.Y., Lin C.H. Neuralangelo: High-fidelity neural surface reconstruction. Proceedings of the IEEE/CVF Conference on Computer Vision and Pattern Recognition.

[18] Wu T., Wang J., Pan X., Xu X., Theobalt C., Liu Z., Lin D. Voxurf: Voxel-based Efficient and Accurate Neural Surface Reconstruction. Proceedings of the International Conference on Learning Representations.

[19] Lin C.H., Ma W.C., Torralba A., Lucey S. BARF: Bundle-adjusting neural radiance fields. Proceedings of the IEEE/CVF International Conference on Computer Vision.

[20] Park K., Henzler P., Mildenhall B., Barron J.T., Martin-Brualla R. (2023). CamP: Camera preconditioning for neural radiance fields. ACM Trans. Graph. (TOG).

[21] Wang P., Zhao L., Ma R., Liu P. BAD-NeRF: Bundle adjusted deblur neural radiance fields. Proceedings of the IEEE/CVF Conference on Computer Vision and Pattern Recognition.

[22] Wang Z., Wu S., Xie W., Chen M., Prisacariu V.A. (2021). NeRF–: Neural radiance fields without known camera parameters. arXiv.

[23] Deng K., Liu A., Zhu J.Y., Ramanan D. Depth-supervised NeRF: Fewer views and faster training for free. Proceedings of the IEEE/CVF Conference on Computer Vision and Pattern Recognition.

[24] Wu R., Mildenhall B., Henzler P., Park K., Gao R., Watson D., Srinivasan P.P., Verbin D., Barron J.T., Poole B. ReconFusion: 3D reconstruction with diffusion priors. Proceedings of the IEEE/CVF Conference on Computer Vision and Pattern Recognition.

[25] Yang J., Pavone M., Wang Y. FreeNeRF: Improving few-shot neural rendering with free frequency regularization. Proceedings of the IEEE/CVF Conference on Computer Vision and Pattern Recognition.

[26] Chen H., Li C., Lee G.H. (2023). NeuSG: Neural implicit surface reconstruction with 3D gaussian splatting guidance. arXiv.

[27] Yu M., Lu T., Xu L., Jiang L., Xiangli Y., Dai B. (2024). GSDF: 3DGS meets SDF for improved neural rendering and reconstruction. Adv. Neural Inf. Process. Syst..

[28] Gortler S.J., Grzeszczuk R., Szeliski R., Cohen M.F. (2023). The lumigraph. Seminal Graphics Papers: Pushing the Boundaries, Volume 2.

[29] Levoy M., Hanrahan P. (2023). Light field rendering. Seminal Graphics Papers: Pushing the Boundaries, Volume 2.

[30] Wood D.N., Azuma D.I., Aldinger K., Curless B., Duchamp T., Salesin D.H., Stuetzle W. (2023). Surface light fields for 3D photography. Seminal Graphics Papers: Pushing the Boundaries, Volume 2.

[31] Barron J.T., Malik J. (2014). Shape, illumination, and reflectance from shading. IEEE Trans. Pattern Anal. Mach. Intell..

[32] Nimier-David M., Vicini D., Zeltner T., Jakob W. (2019). Mitsuba 2: A retargetable forward and inverse renderer. ACM Trans. Graph. (TOG).

[33] LeGendre C., Ma W.C., Fyffe G., Flynn J., Charbonnel L., Busch J., Debevec P. DeepLight: Learning illumination for unconstrained mobile mixed reality. Proceedings of the IEEE/CVF Conference on Computer Vision and Pattern Recognition.

[34] Park J.J., Holynski A., Seitz S.M. Seeing the world in a bag of chips. Proceedings of the IEEE/CVF Conference on Computer Vision and Pattern Recognition.

[35] Richter-Trummer T., Kalkofen D., Park J., Schmalstieg D. (2016). Instant mixed reality lighting from casual scanning. 2016 IEEE International Symposium on Mixed and Augmented Reality (ISMAR).

[36] Chen H., He B., Wang H., Ren Y., Lim S.N., Shrivastava A. (2021). NeRV: Neural representations for videos. Adv. Neural Inf. Process. Syst..

[37] Zhang X., Srinivasan P.P., Deng B., Debevec P., Freeman W.T., Barron J.T. (2021). NeRFactor: Neural factorization of shape and reflectance under an unknown illumination. ACM Trans. Graph. (TOG).

[38] Bai Y., Garg N., Roy N. SPiDR: Ultra-low-power acoustic spatial sensing for micro-robot navigation. Proceedings of the 20th Annual International Conference on Mobile Systems, Applications and Services.

[39] Liang R., Chen H., Li C., Chen F., Panneer S., Vijaykumar N. ENVIDR: Implicit differentiable renderer with neural environment lighting. Proceedings of the IEEE/CVF International Conference on Computer Vision.

[40] Meng J., Li H., Wu Y., Gao Q., Yang S., Zhang J., Ma S. Mirror-3DGS: Incorporating Mirror Reflections into 3D Gaussian Splatting. Proceedings of the 2024 IEEE International Conference on Visual Communications and Image Processing (VCIP).

[41] Jiang Y., Tu J., Liu Y., Gao X., Long X., Wang W., Ma Y. GaussianShader: 3D gaussian splatting with shading functions for reflective surfaces. Proceedings of the IEEE/CVF Conference on Computer Vision and Pattern Recognition.

[42] Gao J., Gu C., Lin Y., Li Z., Zhu H., Cao X., Zhang L., Yao Y. (2024). Relightable 3D gaussians: Realistic point cloud relighting with BRDF decomposition and ray tracing. European Conference on Computer Vision.

[43] Cook R.L., Torrance K.E. (1982). A reflectance model for computer graphics. ACM Trans. Graph. (TOG).

[44] Walter B., Marschner S.R., Li H., Torrance K.E. (2007). Microfacet Models for Refraction through Rough Surfaces. Eurographics Symposium on Rendering.

[45] Karis B., Games E. (2013). Real shading in unreal engine 4. Proc. Phys. Based Shading Theory Pract..

[46] Wizadwongsa S., Phongthawee P., Yenphraphai J., Suwajanakorn S. NeX: Real-time view synthesis with neural basis expansion. Proceedings of the IEEE/CVF Conference on Computer Vision and Pattern Recognition.

